# 免疫治疗耐药后组织学转化为不典型类癌的*RET*融合阳性NSCLC病例报道

**DOI:** 10.3779/j.issn.1009-3419.2025.102.14

**Published:** 2025-05-20

**Authors:** Yu ZHANG, Hui ZHANG, Wei ZHONG, Minjiang CHEN, Mengzhao WANG

**Affiliations:** ^1^100730 北京，中国医学科学院，北京协和医学院，北京协和医院呼吸与危重症医学科; ^1^Department of Respiratory and Critical Care Medicine, Chinese Academy of Medical Sciences and Peking Union Medical College, Beijing 100730, China; ^2^100730 北京，中国医学科学院，北京协和医学院，北京协和医院病理科; ^2^Department of Pathology, Peking Union Medical College Hospital, Chinese Academy of Medical Sciences and Peking Union Medical College, Beijing 100730, China

**Keywords:** 肺肿瘤, 免疫耐药, 组织学转化, 不典型类癌, 高选择性RET抑制剂, Lung neoplasms, Immune resistance, Histological transformation, Atypical carcinoid, Selective RET inhibitors

## Abstract

近年来，免疫检查点抑制剂（immune checkpoint inhibitors, ICIs）已成为驱动基因阴性晚期非小细胞肺癌（non-small cell lung cancer, NSCLC）治疗的基石，然而耐药不可避免且机制尚不明确。其中，组织学类型转化是罕见的免疫耐药机制，目前仅有个案报道。本文报道了首例经一线ICIs联合化疗后组织学类型转化为不典型类癌的肺腺癌，探讨了组织类型转化在NSCLC获得性免疫耐药机制中的关键作用。此外，患者携带转染重排基因（rearranged during transfection, *RET*）融合突变，在发生组织类型转化后试用塞普替尼治疗取得了良好的疗效，本例为合并罕见突变的不典型类癌患者提供了可能的药物治疗选择。

## Abstract (en)

Immune checkpoint inhibitors (ICIs) have become the cornerstone of treatment for driver gene-negative advanced non-small cell lung cancer (NSCLC). However, resistance is inevitable, and the underlying mechanisms remain incompletely understood. Histological transformation is a rare but emerging cause of acquired resistance to immunotherapy, with only sporadic case reports documented to date. Here, we report the first case of lung adenocarcinoma that underwent histological transformation to atypical carcinoid following first-line therapy with ICIs combined with chemotherapy, highlighting the critical role of histological lineage switching in mediating NSCLC resistance to ICIs. Notably, the patient harbored a rearranged during transfection (*RET*) fusion mutation. Subsequent targeted therapy with Selpercatinib after histological transformation demonstrated favorable efficacy, suggesting a potential therapeutic strategy for atypical carcinoid patients with co-occurring rare driver mutations. This case provides a potential therapeutic option for atypical carcinoid patients with rare mutations.

近年来，免疫检查点抑制剂（immune checkpoint inhibitors, ICIs），特别是程序性细胞死亡受体1（programmed cell death 1, PD-1）/程序性细胞死亡配体1（programmed cell death ligand 1, PD-L1）抑制剂，已经显著改善了晚期非小细胞肺癌（non-small cell lung cancer, NSCLC）患者的预后^[1,2]^，成为国内外指南推荐的一线标准治疗，但耐药仍是临床中不可避免的挑战。目前报道的导致获得性ICIs耐药机制多种多样，可以大致分为以下3类：（1）肿瘤细胞相关机制，包括新抗原丢失、新抗原呈递缺陷以及肿瘤内部新抗原及新突变；（2）信号传导缺陷，包括通过癌基因信号传导导致T细胞排斥，以及转移灶对T细胞诱导的溶解产生耐药性；（3）肿瘤微环境相关机制，包括转移灶的位置、多种免疫抑制调节因子的表达、细胞毒性T细胞耗竭、免疫抑制性微环境的形成以及变化等，这些机制往往同时存在^[3,4]^。其中，组织类型转化虽不常见，但正逐渐受到关注。由于临床医生往往忽视免疫治疗耐药后的再次活检，组织学转化的发生率仍有待在大样本和真实世界临床研究中得到证实。

携带转染基因重排（rearranged during transfection, *RET*）融合突变是NSCLC中一种较为罕见的驱动基因突变，发生率为1%-2%。尽管如此，由于肺癌患者群体基数庞大，携带此类突变的患者不在少数。迄今为止，已发现至少48个与*RET*融合相关的伴侣基因。其中，*KIF5B-RET*和*CCDC6-RET*是最常见的融合类型。还有一些较为罕见的融合类型，如*NCOA4-RET*、*ERC1-RET*和*TRIM24-RET*等^[5]^。

本文报道了1例携带*RET*融合突变的晚期NSCLC患者。在确诊时，由于高选择性RET抑制剂（selective RET inhibitors, SRIs）的可及性受限，该患者未能接受靶向治疗，而接受了免疫联合化疗的治疗方案，得到了长期病情缓解，但随后病情进展，再次组织活检提示组织学转化为不典型类癌（atypical carcinoid, AC）。尽管肿瘤组织类型发生改变，但*RET*融合突变依然存在，患者后续接受SRIs治疗并取得显著临床疗效。本文探讨了组织类型转化作为免疫耐药机制之一的重要性，以及SRIs在组织类型转化后的有效性，为临床治疗提供了新的思路。

## 1 病例资料

患者，男性，66岁，2020年3月因“右侧胸痛2周”就诊于北京协和医院门诊。高血压病史10年余，长期大量吸烟史（吸烟40年，每日约10支）。体格检查如下：血压135/78 mmHg，心率72次/min，体温36.5 ^o^C，脉搏血氧饱和度98%（未吸氧状态下）。身高170 cm，体重68.5 kg，体重指数（body mass index, BMI）23.7 kg/m^2^，美国东部肿瘤协作组体能状态（Eastern Cooperative Oncology Group performance status, ECOG PS）评分1分。右颈部可触及数个小淋巴结，质韧，活动度尚可，双下肺可闻及爆裂音，右肺呼吸音减低，未闻及湿啰音。心脏听诊律齐。腹软，无压痛，双下肢无水肿。行胸部电子计算机断层扫描（computed tomography, CT）示右肺上叶实性占位（4.9 cm×4.7 cm），伴纵隔及右锁骨上淋巴结肿大，右侧胸腔及心包少量积液。完善全身骨显像及头颅增强磁共振成像（magnetic resonance imaging, MRI）排除骨转移及中枢神经系统转移。2020年4月于外院行右上纵隔淋巴结穿刺活检（初诊时淋巴结转移灶活检），病理示恶性肿瘤细胞浸润考虑为癌巢浸润，结合免疫组化结果为腺癌浸润，鉴于甲状腺转录因子1（thyroid transcription factor-1, TTF-1）（+），临床重点检查肺脏，免疫组化：细胞角蛋白7（cytokeratin 7, CK7）（+），TTF-1（+），P63（-），P40（-），CK5/6（-），Ki-67（增殖指数：65%），新天冬氨酸蛋白酶（new aspartic proteinase A, Napsin A）（-），CD56（-），突触核蛋白（synuclein, Syn）（-），嗜铬粒蛋白A（chromogranin A, CgA）（-）， 波形蛋白（Vimentin）（部分+）。穿刺组织标本基因检测：表皮生长因子受体（epidermal growth factor receptor, EGFR）（-），*KIF5B-RET*基因融合突变，丰度14.85%，RB1外显子6错义突变，丰度22.79%，*TP53*外显子7错义突变，丰度12.65%。根据美国癌症联合委员会（American Joint Committee on Cancer, AJCC）第8版分期为cT2bN3M1a，IVA期。2020年5月起，患者接受了4个周期的诱导治疗，方案为培美曲塞900 mg联合卡铂500 mg（每21天为1个周期，每个周期第1天予以上述药物静脉输液治疗），同时，每个周期中患者还联合应用帕博利珠单抗200 mg。诱导治疗结束后，患者自述胸痛症状明显减轻，ECOG PS评分0分。后继续接受共26个周期维持治疗，方案为培美曲塞900 mg联合帕博利珠单抗200 mg（每21天为1个周期，每个周期第1天予以上述药物静脉输液治疗）。治疗过程中评估最佳疗效为部分缓解（右肺上叶占位1.9 cm×1.7 cm）。2023年3月起患者停止抗肿瘤治疗，定期门诊随诊。2023年10月患者因再次出现右侧胸痛，复查胸部CT示原病灶增大，未发现新病灶。2023年12月于外院行右肺原发灶处穿刺活检（复发时肺内原发灶活检），病理示AC，免疫组化：AE1/AE3（+++），CK7（+++），TTF-1（+++），CgA（+++），Syn（+++），CD56（+++），Napsin A（-），P40（-），间变性淋巴瘤激酶（anaplastic lymphoma kinase, ALK）（-），Ki-67（增殖指数：20%+）。PD-L1：肿瘤细胞阳性比例分数（tumor proportion score, TPS）<1%。肺组织基因检测：*KIF5B-RET*基因融合突变，丰度21.19%。患者因经济原因未应用SRIs，2024年2月起接受共4个周期化疗治疗，方案为依托泊苷0.17 g联合卡铂400 mg（每21天为1个周期，在每个周期第1天予以上述药物静脉输液治疗），后复查胸部CT示病灶继续增大（3.9 cm×3.9 cm），评估病情进展。2024年6月起调整化疗方案，方案为多西他赛110 mg（每21天为1个周期，每个周期第1天予以上述药物静脉输液治疗），因III度骨髓抑制，仅接受1个周期该药治疗。2024年8月，患者调整治疗方案为塞普替尼160 mg *bid*，口服。治疗期间出现I度白细胞减低、II度血小板下降及中度肝功能异常，将塞普替尼减量至120 mg bid，口服，且予以对症治疗后，血象及肝功能逐渐恢复至正常。患者自述胸痛症状逐渐缓解并消失，ECOG PS评分0分，胸部可见轻度皮肤脱屑。后患者规律用药，2025年3月复查胸部CT示原发灶缩小，再次评估为部分缓解（图1）。

**图 1:**
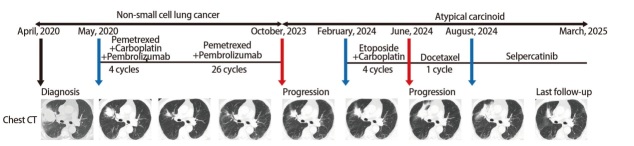
该患者的治疗经过以及影像学表现

## 2 讨论

尽管ICIs在晚期NSCLC患者中展现出显著疗效，但临床实践中仅少部分患者获得持久应答^[6]^。免疫治疗获得性耐药的机制尚未完全明确，肿瘤细胞和肿瘤微环境在免疫治疗期间的适应性变化是产生耐药的关键机制。目前报道ICIs的耐药机制包括：肿瘤内源性因素如表皮生长因子受体（epidermal growth factor receptor, *EGFR*）、ALK等驱动基因的活化，人类第10号染色体缺失的编码与磷酸酶和张力蛋白同源的基因（phosphatase and tensin homologue deleted on chromosome 10, *PTEN*）、丝氨酸/苏氨酸蛋白激酶11（serine-threonine kinase 11, *STK11*）等抑癌基因的失活，以及克隆选择、表观遗传抑制导致新抗原丢失，这些因素导致免疫逃逸引发肿瘤进展；肿瘤外源性因素如T细胞功能障碍、免疫抑制性细胞形成免疫抑制性微环境，进而引发免疫抵抗。此外，宿主因素如性别、种族、微生物组及饮食等也可能影响免疫治疗的反应，从而导致获得性耐药^[7,8]^。

组织类型转化是一种少见的耐药机制，是肿瘤谱系可塑性的典型体现。在*EGFR*突变的NSCLC患者中，有3%-14%的患者在接受EGFR-酪氨酸激酶抑制剂（EGFR-tyrosine kinase inhibitors, EGFR-TKIs）靶向治疗后会出现小细胞肺癌（small cell lung cancer, SCLC）的转化^[9,10]^。既往研究提出两种假设，其中一种为组织学类型转化假说，即NSCLC在特定条件下发生谱系重编程，如*EGFR*突变、*RB1*和*TP53*失活、*SMAD4*缺失以及*MYC*扩增等，直接转化为神经内分泌肿瘤（neuroendocrine neoplasms, NENs）亚型^[11]^。目前已知*TP53*与*RB1*的协同失活突变是触发神经内分泌转化的关键前提条件^[12]^，也有研究^[13]^证实，*SMAD4*的缺失可以通过上调*ASCL1*基因的转录，加速NSCLC的神经内分泌转化，然而，这一转化可能是一个由多个基因组改变、转录变化和表观遗传学重塑的逐步过程^[14]^。另一种为克隆选择假说，即初始肿瘤可能已包含NSCLC和NENs的混合成分，在治疗后NENs成为优势成分表现为组织类型转化，此类机制在初诊病理已显示神经内分泌标志物表达的患者中更为常见^[11]^。

然而，在接受免疫治疗的患者中，组织类型转化仅有个案报道，其总体发生率和具体机制尚不明确。本文搜索PubMed、Web of Science数据库并总结免疫治疗后转化为SCLC^[15-25]^的病例报道，见表1。部分患者在诊断时即出现了神经内分泌成分，提示免疫治疗后优势成分的改变可能是组织类型转化的机制。本例患者治疗前病理组织未显示神经内分泌分化特征，疾病进展后二次活检证实组织学类型转化为AC。虽然两次活检均为小标本穿刺，可能无法明确除外本身存在NENs成分的可能，但患者两次活检标本均存在*KIF5B-RET*融合突变，提示更可能为谱系重编程相关的组织类型转化。其中*RET*融合突变在组织学类型转化中的分子机制仍需进一步研究阐明。

**表 1:** 免疫治疗后出现组织学转化的病例

Reference	Age of initial diagnosis (yr)/ Gender	Set of first biopsy	Histology	Immunotherapy	The biopsy site after the progression	Histology after progression	Genomic profile
Imakita T, 2017^[15]^	75/M	Lung	NSCLC	Nivo	Pleural effusion/ Subcutaneous tumor	SCLC/SCLC	No
Abdallah N, 2018^[16]^	65/M	Pleural effusion	Ad	Nivo	Lung	SCLC	No
	68/M	Lung	NSCLC	Pembro	LN (mediastinum)	SCLC	No
Okeya K, 2019^[17]^	66/M	Liver	Ad	Pembro	Pleural effusion	SCLC	No
Bar J, 2019^[18]^	70/F	Lung	Sq with NE features	Nivo	Adrenal gland	SCLC	TP53
	75/M	Lung	Sq with NE features	Nivo	Lung	SCLC	No
Iams WT, 2019^[19]^	67/F	Lung	Ad	Nivo	Pleural effusion/Pericardial effusion	SCLC/SCLC	No
	75/F	Lung	Ad	Nivo	LN (mediastinum)	SCLC	KRAS G12C
Sehgal K, 2020^[20]^	60/F	Lung	Sq	Nivo	Lung/LN (mediastinum)	SCLC/SCLC	TP53
Si X, 2020^[21]^	69/M	Lung	Sq	Nivo	Pleural effusion/Lung	SCLC/SCLC	NA
Imakita T, 2021^[22]^	64/M	Lung	Sq	Nivo	Adrenal gland/Lung	LCNEC/SCLC	No
	74/F	Lung	Sq	Nivo	Chest wall	SCLC	No
Liu H, 2022^[23]^	75/M	Lung	Sq	Nivo	Lung	SCLC	NA
Wang D, 2023^[24]^	56/M	Lung	Sq	SGM	LN (mediastinum)	SCLC	NA
Li Q, 2024^[25]^	67/F	Lung	Sq	SNTL	Lung	SCLC	NA

M: Male; F: Female; NSCLC: non-small cell lung cancer; SCLC: small cell lung cancer; LCNEC: large cell neuroendocrine carcinoma; NE: neuroendocrine; Sq: squamous cell carcinoma; Ad: adenocarcinoma; Nivo: Nivolumab; Pembro: Pembrolizumab; SGM: Sugemalimab; SNTL: Sintilimab; LN: lymph node; NA: not available.

此外，现行指南明确推荐对进展期NSCLC患者进行二次活检，尤其是在靶向治疗耐药后^[26]^。在临床工作中，对于NSCLC靶向治疗后病情进展的患者，医生通常会积极进行活检以明确是否存在组织类型转化，同时借助基因检测了解耐药基因情况。然而，在免疫治疗失败后，医生往往忽视了这一步骤，这可能导致组织类型转化的发生率在真实世界中被明显低估。本文旨在强调免疫治疗耐药后二次活检在监测肿瘤演变和指导后续治疗中的重要性。

在LIBRETTO-001、ARROW I/II期研究中SRIs后线治疗*RET*融合突变NSCLC的中位无进展生存期（median progression-free survival, mPFS）分别为17.1和24.9个月^[27,28]^。本例患者存在*RET*融合突变，但治疗初期由于药物可及性等客观因素未能一线应用SRIs，但在病情进展后，后线应用塞普替尼依然显示良好的疗效，PFS目前已达7个月，且患者仍在规律随诊中，这提示即便出现组织类型转化为AC，使用SRIs靶向治疗仍能对*RET*阳性的患者带来良好的疗效。结合既往塞普替尼在*RET*融合阳性AC患者中的有效性个案研究报道^[29]^，本病例也进一步拓展了对*RET*阳性肺癌患者靶向治疗的认识以及SRIs在不同组织学类型肺癌中的应用前景。

本文报道了1例携带*RET*融合突变的晚期NSCLC患者，在接受ICIs联合化疗治疗后，得到长期疾病缓解后出现免疫耐药，进一步检查发现，患者的肿瘤组织学类型转化为AC。尽管肿瘤组织学类型发生改变，但*RET*融合突变依然存在，后续接受塞普替尼治疗并取得显著临床疗效。这一发现不仅进一步证实了塞普替尼在AC患者中的有效性，还强调了组织学类型转化作为免疫耐药机制的重要性，以及在免疫治疗耐药后进行二次活检的必要性。
